# Descriptive epidemiology of objectively-measured, free-living sleep parameters in a rural African setting

**DOI:** 10.1186/s13104-020-05153-8

**Published:** 2020-07-01

**Authors:** Ian Cook, Matlawa Mohlabe, Marianne Alberts

**Affiliations:** 1 Physical Activity Epidemiology Laboratory (EDST), University of Limpopo (Turfloop Campus), Sovenga, PO Box X1106, Limpopo 0727 South Africa; 2 Department of Pathology and Medical Sciences, University of Limpopo (Turfloop Campus), Sovenga, Limpopo South Africa

**Keywords:** Body composition, Anthropometry, Accelerometer, Actigraphy, Movement monitor, Measurement

## Abstract

**Objectives:**

To investigate the descriptive nature of objectively-measured, free-living sleep quantity and quality, and the relationship to adiposity, in a rural African setting in 145 adults (≥ 40 years, female: n = 104, male: n = 41). Wrist-mounted, triaxial accelerometry data was collected over 9 days. Measures of sleep quantity and quality, and physical activity were extracted from valid minute-by-minute data. Adiposity indices were body-mass-index, waist circumference and conicity index. Self-reported data included behavioural, health and socio-demographic variables. Community consultation followed the quantitative data analyses, for validation and interpretation of findings.

**Results:**

Females had more nocturnal sleep than males (7.2 vs. 6.8 h/night, *p *= 0.0464) while males recorded more diurnal sleep time (*p *= 0.0290). Wake after sleep onset and number of awakenings were higher in females, and sleep efficiency was higher in males (*p *≤ 0.0225). Sleep indices were generally similar between weekdays and weekends, except for sleep fragmentation index (*p *= 0.0458). Sleep quantity, but not sleep quality was independently and inversely associated with adiposity (*p *= 0.0453). Physical activity and morbidity measures were significantly and consistently associated with sleep and adiposity measures (*p *< 0.0458). The preliminary qualitative data suggests that future studies should include more detailed data around contextual issues of sleep (social, cultural, economic, environment).

## Introduction

Sleep is recognized as an important lifestyle contributor to morbidity and mortality [1]. While extensive Sleep Health Epidemiology literature exists for high income countries, there is a need of especially objective measures of sleep, such as actigraphy, from low and middle-income countries, [2, 3]. Within the South African context to date, studies have exclusively utilized self-report measures in adult populations [4–12]. Hence, there is a dearth of objectively-measured sleep data from South African, and specifically rural African settings [3, 13]. Self-reported long sleep time for rural South Africans [8–10], requires confirmation using objective measures of sleep duration. A recent study found a significant, inverse relationship between self-reported sleep duration and adiposity in a rural African setting [14]. Given the lack of objectively-measured, free-living sleep parameters in any South African setting, the objective of this study was to use wrist-actigraphy to derive sleep parameters in a rural African setting during a cross-sectional survey, and thus extend the findings of self-report sleep duration and the relationship to adiposity [4–12, 14].

## Main text

### Methods

#### Dikgale health and demographic surveillance system site (DHDSS) sample [15]

A convenience sample of 167 adult participants was recruited from the AWI-Gen Phase 1 Study cohort (≥ 40 years), the methodological details of which are reported in detail elsewhere [14, 16]. Trained field workers collected self-reported, measured and biological data from participants by means of questionnaires, anthropometry, ultrasound scans, and venipuncture [14, 16]. We calculated body mass index (BMI, kg/m^2^) and conicity index (CI) [17]. The latter measure allows a single-measure of body shape to be used in multivariate analyses. Questionnaire data included behavioural, health and socio-demographic variables and is reported in detail elsewhere [14, 16]. The ultrasound scans and blood-derived variables were not considered for this analysis.

#### Accelerometer data collection and data reduction

Participants wore a small, light-weight, wrist-worn triaxial accelerometer for 9 days (ActiGraph wGT3X-BT, Actigraph, LLC, Pensacola, FL, 2013) [18–21]. Prior to use, the monitors were connected to an IBM-compatible computer via USB interface and initialized to sample at 30 Hz, using proprietary software (Actilife 6.13.4, Actigraph, LLC, Pensacola, FL, 2009–2015). The monitors were affixed with a proprietary woven nylon wristband on the non-dominant wrist, and unless there was to be a sustained period of water immersion, participants were requested not to remove the monitors. On the 9th day, the monitors were collected from the participants. Thereafter, the raw recorded data was downloaded from the monitors onto an IBM-compatible computer and stored for later analysis. Prior to re-use of the monitors, the batteries were fully recharged and the memory cleared of previous data. The wrist straps were washed using a disinfectant solution, rinsed in water and air-dried.

Using proprietary software (Actilife 6.13.4, Actigraph, LLC, Pensacola, FL, 2009–2015), valid data (at least 1weekday and 1 weekend day) was obtained by first converting downloaded, raw data files to 60 s epochs. Thereafter, the Cole-Kripke sleep scoring algorithm was used to determine minute-by-minute asleep/awake status [22], and the Actilife-modified Tudor-Locke algorithm to identify sleep periods [23, 24]. Valid wear-time was evaluated using the Choi algorithm [25, 26], with sleep time marked as wear time, and a valid day requiring ≥ 10 h of wear time. Vector Magnitude (VM)- and Ambulation-defined physical activity variables were defined as counts/day and counts/minute, and steps/day and steps/minute, respectively [27]. Sleep indices included Total Sleep Time (TST), Sleep Efficiency (SE), Wake After Sleep Onset (WASO) and Sleep Fragmentation Index (SFI) [28–30].

Valid physical activity and sleep data was downloaded in a summarised and detailed format in Microsoft Excel™ files and additional variables were extracted: diurnal and nocturnal sleep time, sleep periods during defined hours, and sleep variation across days (within-person total sleep time SD). Diurnal and nocturnal periods were defined as 06h00–18h00 and 18h01–05h59, respectively. The number of sleep periods initiated between 00h01 and 05h59 defined an additional sleep quality indicator. A sleep period falling completely within the period 06h00–18h00 was defined as potentially a “daytime napping” period. Sufficient sleep quantity and quality were defined as 7–9 h [31] and SE ≥ 85%, respectively [32]. Data was then imported into statistical software for further analyses.

#### Statistical analyses

Descriptive statistics comprised means (one standard deviation), medians (interquartile range) and frequencies. Relationships between categorical variables were examined through the Chi square Test. For continuous data, independent and dependent *t*- tests examined differences between the sexes and weekday/weekend days and where required, the appropriate non-parametric test was employed. Due to non-normality, continuous variables were transformed to quantiles as required. Bivariate relationships were examined using correlation coefficients.

Multiple linear regression models were examined for predictors of sleep indices (TST, SE, WASO, SFI) and body composition measures (waist circumference and BMI) using selected socio-demographic, behavioural and biological variables. Forced and Backward selection (*p* in = 0.05, *p* out = 0.10) models were employed. Separate sex-specific analyses were run, specifically to include parity in the female analysis. For multivariate adiposity analyses TST and SE were used in forced models, while TST, SE, WASO, SFI were entered in selection models.

To analyze the trend across days for objectively-measured sleep indices, the day-by-day data was analysed by fitting a mixed-effects model (Fixed effect, Type III, Restricted Maximum Likelihood), using a compound symmetry covariance matrix. Missing values were considered missing completely at random. The Geisser-Greenhouse correction was adopted throughout. Multiple comparisons tests (Tukey) compared sleep indices across each day.

Data were analysed using appropriate statistical software (IBM SPSS Statistics: Release 25 IBM Corporation, Armonk NY, 2017 and GraphPad Prism: version 8.3.0, GraphPad Software, La Jolla CA, 2019). Significance for all inferential statistics was set at *p *< 0.05.

#### Informant consultation

To add contextual detail to the quantitative results, we obtained feedback from DHDSS fieldworkers (n = 2) and the community engagement officer through an interview [33]. Key findings were discussed and informants were encouraged to provide feedback, which was captured via notes. After the interview, the notes were distributed for confirmation.

## Results

Of the 167 raw data files, 157 had valid data for at least one weekday and one weekend day. Once combined with the questionnaire and body composition data, 145 had complete data. Participants averaged 7.54 (0.61) days of valid data, with 94.3% (7.6) wear time and 5.7% (7.6) of non-wear time. The number of weekdays and weekend days with valid data was 5.9 (0.5) and 2.0 (0.2) days, respectively. Except for self-reported PA (*p *= 0.002), there were no significant differences in socio-demographic, behavioural or biological variables between the study participants and the full DHDSS AWI-Gen sample [14]. There was no significant difference between participants with 7 days (n = 90) versus < 7 days (n = 55) of accelerometry data across socio-demographic, behavioural and biological variables (p ≥ 0.128), except for fruit and vegetable intake (p = 0.013).

A total of 1148 sleep periods was recorded, of which 13 (1.1%) fell completely within the period 06h00-18h00. Four of these sleep periods were recorded by HIV + participants. Three of these four periods (309-340 min, 5-14 awakenings, WASO 28-57 min) were recorded by one HIV + male (56 years, 19.2 kg/m^2^).

In a comparison between HIV + and HIV- participants, only WASO (55 vs. 49 min, respectively) and number of awakenings (NOA) (16 vs. 14 awakenings, respectively) were significantly different (*p *≤ 0.0404).

Females displayed significant levels of adiposity while no males were obese (*p *< 0.0001) (Table 1). Females also displayed higher prevalence of hypertension, consumption of sugar-sweetened beverages, and levels of objectively measured PA (*p *≤ 0.0050). However, significantly more males than females reported current use of alcohol (*p *< 0.0001) (Table 1).

**Table 1:** Descriptive statistics of demographic, behavioural and biological characteristics by sex

	All (n = 145)	Female (n = 104)	Male (n = 41)	*P* value
**Socio-demographic**				
Age (years)	52.6 (7.0)	52.1 (6.7)	54.1 (7.5)	0.1455
Marital status (Married/Co-habiting)^b^	55.9 (81)	55.8 (58)	56.1 (23)	0.6342
Level of education (formal education)^b^	95.2 (138)	95.2 (00)	95.1 (39)	0.9966
Employed (Yes)^b^	26.2 (38)	26.0 (27)	26.8 (11)	0.9148
SES Quintile	3.6 (1.3)	3.6 (1.3)	3.5 (1.3)	0.5496
Housing density^a^				
People/room	0.8 (0.8)	0.9 (0.7)	0.7 (0.7)	*0.0430*
People/bedroom	1.5 (1.3)	1.7 (1.3)	1.3 (1.0)	*0.0280*
**Behavioural**				
Diet^a^				
Fruit and vegetable intake (servings/day)	1.3 (0.9)	1.3 (1.2)	1.1 (0.4)	0.0710
Sugar sweetened beverages (servings/day)	0.3 (0.1)	0.3 (0.1)	0.3 (0.0)	*0.0050*
Tobacco use (current smoke and/or smokeless; Yes)	48.3 (70)	45.2 (47)	56.1 (23)	0.2366
Alcohol use (current; Yes)	20.0 (29)	8.7 (9)	48.8 (20)	*< 0.0001*
Self-reported physical activity^a^				
MVPA (minutes/week)	990 (968)	1020 (1005)	840 (1170)	0.2740
Self-reported sleep				
Sleep (hours/night)	9.2 (1.6)	9.1 (1.4)	9.5 (1.8)	0.1924
Weekday sleep (hours/night)	8.9 (1.7)	8.7 (1.5)	9.4 (2.0)	0.0694
Weekend sleep (hours/night)	9.8 (2.2)	9.8 (2.3)	9.7 (1.8)	0.8137
Objectively-measured physical activity				
VM counts/day (x 10^6^)	2.33 (0.70)	2.47 (0.63)	1.99 (0.76)	*0.0007*
VM counts/minute	1621 (486)	1715 (435)	1383 (530)	*0.0007*
Steps per day	14,416 (4637)	14,140 (3718)	15,116 (6411)	0.3645
Step cadence (steps/minute)	10.0 (3.2)	9.8 (2.6)	10.5 (4.5)	0.3652)
**Biological**				
Waist circumference (cm)	90.0 (1.6)	93.5 (1.6)	81.2 (10.0)	*< 0.0001*
Body mass index (kg/m^2^)	28.1 (7.8)	30.6 (7.6)	21.9 (3.9)	*< 0.0001*
Body mass index categories^b^				
Under- normal weight (< 25 kg/m^2^)	37.2 (54)	24.1 (25)	70.7 (29)	*< 0.0001*
Overweight (25–29.99 kg/m^2^)	24.1 (35)	22.1 (23)	29.3 (12)	
Obese (≥ 30 kg/m^2^)	38.6 (56)	53.8 (56)	0.0 (0)	
HIV status (Yes)^b^	22.1 (32)	21. (22)	24.4 (10)	0.6722
Hypertension (Yes)^b^	50.3 (73)	55.8 (58)	36.6 (15)	*0.0375*
Diabetes mellitus (Yes)^b^	4.1 (6)	3.8 (4)	4.9 (2)	0.5451
Parity	–	4.2 (1.8)	–	

Italicized* p* values are significant (*p* < 0.05)

Data reported as mean (SD)

*HIV* Human Immunodeficiency Virus, *MVPA* Moderate to vigorous physical activity, *SES* Socio-economic status, *VM* Vector magnitude

^a^Median (IQR) or ^b^% (n)

Sleep quantity (nocturnal) was significantly higher in females (*p *≤ 0.0464), while sleep quality indices were significantly poorer in females (*p *≤ 0.0290) (Table 2). Males slept more during the day (*p *= 0.0290), were in bed more than 90 min earlier than females (*p *= 0.0016) and had significantly more sleep periods starting between 00:01-05:59 (*p *= 0.0084) (Table 2). There was no association between sufficient sleep categories for TST or nocturnal sleep time, and gender or day of the week (*p *≥ 0.4904) (Table 2).

**Table 2:** Objective measures of sleep quantity and -quality indices across sex and day of the week

	Sex			Day of the week		P-values
	Female (n = 104)	Male (n = 41)	P-values	Weekday (n = 145)	Weekend (n = 145)	
**Quantity**						
Sleep time (minutes/day)						
Total	458 (67)	454 (105)	0.8175	456 (86)	458 (91)	0.6828
Diurnal^a^	15 (37)	20 (59)	0.1560	10 (43)	14 (39)	0.6110
Nocturnal	433 (60)	410 (72)	*0.0464*	427 (70)	427 (76)	0.9380
Nocturnal/Total sleep (%)	95.0 (5.7)	91.6 (9.9)	*0.0463*	94.4 (7.7)	93.8 (8.9)	0.3553
Diurnal sleep time ≥ 1 min^c^						
Prevalence^b^	83.7 (87)	80.5 (33)	0.6495	54.0 (102)	46.0 (87)	0.0645
Duration (minutes)^a^	56 (71)	69 (73)	*0.0290*	55 (73)	64 (64)	–
Sleep period starting between 00:01 and 05:59^b^	34.6 (36)	58.5 (24)	*0.0084*	31.0 (45)	20.7 (30)	*0.0443*
Sleep period between 06:00 and 18:00^b^	5.8 (6)	9.8 (4)	0.3935	4.1 (6)	4.1 (6)	1.0000
Clock time (hh:mm)						
In bed	19:50 (2:03)	18:14 (2:49)	*0.0016*	19: 19 (2:53)	19:31 (3:40)	0.5725
Out of bed	5:18 (1:10)	5:50 (2:13)	0.1544	5:28 (1:50)	5:29 (1:43)	0.8690
Sleep latency (minutes)^a^	0.3 (0.7)	0.5 (0.7)	0.9440	0.4 (0.8)	0.3 (1.0)	0.6890
Sufficient sleep categories^b^						
Total sleep time						
<7 h/day	33.7 (35)	41.5 (17)	0.6669	35.9 (52)	40.0 (58)	0.7647
7–9 h/day	51.0 (53)	43.9 (18)		47.6 (69)	44.1 (64)	
>9 h/day	15.4 (16)	14.6 (6)		16.6 (24)	15.9 (23)	
Nocturnal sleep time						
<7 h/night	46.2 (48)	56.1 (23)	0.4904	47.6 (69)	44.8 (65)	0.6954
7–9 h/night	50.0 (52)	39.0 (16)		48.3 (70)	49.0 (71)	
>9 h/night	3.8 (4)	4.9 (2)		4.1 (6)	6.2 (9)	
**Quality**						
Wake after sleep onset (minutes)	53 (16)	44 (17)	*0.0022*	51 (18)	50 (20)	0.8219
Number of awakenings	15 (4)	12 (4)	*0.0011*	14 (5)	14 (5)	0.9577
Average awakening length (minutes)	3.8 (1.0)	4.1 (1.6)	0.3339	4.0 (1.4)	3.9 (1.5)	0.5015
Sleep efficiency (%)	87.7 (4.2)	89.6 (4.5)	*0.0225*	88.1 (4.6)	88.4 (4.9)	0.2440
Achieved ≥ 85% ^b^	79.8 (83)	82.9 (34)	0.6683	75.9 (110)	76.6 (111)	0.8903
Within-person sleep time SD (minutes)^a^	85.1 (48.5)	90.6 (80.9)	0.2480	84.3 (60.8)^d^	66.2 (63.2)^d^	–
Movement index (%)	15.5 (3.8)	16.6 (3.6)	0.1266	16.0 (4.0)	15.5 (4.4)	0.0617
Fragmentation index (%)	11.0 (4.4)	11.3 (4.2)	0.7219	11.4 (4.7)	10.7 (5.7)	0.1755
Sleep fragmentation index (%)	26.6 (7.1)	27.9 (6.8)	0.2986	27.4 (7.4)	26.2 (8.7)	*0.0458*
VM average counts	34,847 (12,090)	27,495 (10,686)	*0.0006*	33,042 (13,162)	31,966 (13,767)	0.2951

Italicized* p* values are significant (*p* < 0.05)

Data reported as mean (SD)

*SD* standard deviation, *VM* Vector Magnitude

^a^Median (IQR) or ^b^ % (n);^c^ n differs from column totals; ^d^n differs from column totals 142 and 141, respectively; Diurnal: 06h00–18h00; Nocturnal: 18h01–05h59

Sleep quality (SFI) was significantly poorer on weekdays (*p *= 0.048) and significantly more sleep periods starting between 00:01-05:59 (*p *= 0.0443) occurred on weekdays (Table 2).

There was no significant association between self-reported TST (Table 1) and objectively-measured TST (Table 2) (*p *≥ 0.0568).

**Fig. 1:**
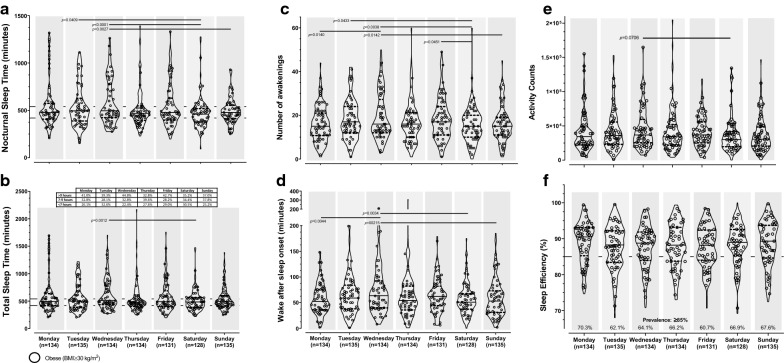
Accelerometry-derived sleep-quantity and -quality measures across days of the week. **a** Nocturnal sleep time (minutes); **b** Total sleep time (minutes); **c** Number of awakenings; **d** Wake after sleep onset (minutes); **e** Activity counts during sleep time; **f** Sleep efficiency (percentage). The day-by-day sleep indices are presented using violin plots (medium smoothing). The horizontal lines above the day of the week indicate significant post hoc differences (Tukey procedure) between days at either end of the line

Significantly more nocturnal sleep time was accrued on Tuesday and Wednesday compared with the weekend (*p *≤ 0.0409) (Fig. 1A), and a similar pattern was found for TST (Fig. 1B). Less than 40% of the participants slept 7-9 h (Fig. 1B). Generally NOA and WASO were significantly higher on Tuesday-Wednesday, compared with the Saturday-Monday (*p *≤ 0.0451) (Fig. 1C-D). Activity counts during sleep were significantly higher on Wednesday compared with Saturday (*p *= 0.0076) (Fig. 1E) and 60-70% of participants achieved SE ≥ 85% (Fig. 1F). There were no significant differences across days for diurnal sleep time (*p *= 0.3463), any fragmentation index (*p *≥ 0.2828), or length of awakenings (*p *= 0.3463).

Except for TST, sleep indices were not associated with adiposity measures in multivariate analyses (Additional file 1: Tables S1–S2, see Additional file 1). In contrast, current morbidity status, socio-demographic indices and lifestyle factors were significantly associated with adiposity indices (Additional file 1: Table S1-S2, see Additional file 1). In female models, parity was not related to adiposity level (*p *> 0.05).

PA was significantly associated with TST and SE (*p *≤ 0.0077), while socio-economic and -demographic indices were significantly associated with sleep indices (*p *≤ 0.0389) (Additional file 1: Table S3-S4, see Additional File 1). In female models, parity was significantly associated with TST and SE (*p *≤ 0.0430) (Additional file 1: Table S4, see Additionalf 1).

PA was significantly related to WASO and SFI in forced and backward-selection sleep models (*p *≤ 0.0360).

The qualitative results provide insight into the quantitative trends (see Additional file 2). Health outcomes (adiposity) and sleep patterns were associated with a number of themes; gender roles, cultural preferences/practices and community activities, education and living conditions.

## Discussion

This analysis is novel in that, as far as the authors are aware, this is the first free-living, objectively-measured sleep data from a South African setting.

The main findings of this analysis were first that females tended to sleep more than males, although sleep quality tended to be worse in females. Second, sleep variables were generally similar between weekdays and weekend days. Third, only one sleep variable was associated with measures of adiposity, while PA variables were significantly related to adiposity and sleep variables. Fourth, morbidity measures were consistently associated with adiposity and sleep variables.

To date, the average self-reported sleep time for black South Africans (male/female, rural/urban) is ± 9 h (range: 8–10.4 h) [4, 6, 8–12, 14]. While some studies have reported gender differences for TST in rural samples (females > males) [4, 14], Peltzer et al. found the reverse (males > females) [8], and others found no gender differences, specifically in urban samples [9, 12]. In contrast, our objective-measure showed approximately 2 h lower TST for both genders compared with self-report measures. In agreement with some self-report studies [4, 14], females recorded more nocturnal sleep time than males.

Our results for sufficient sleep (46.9%) fell within the range reported for self-report measures (38–64.2%) [4, 6, 7, 10, 28]. In contrast, we found a higher prevalence for short sleep 49.0% vs. 5.7–20.8%) and a lower prevalence for long sleep (4.1% vs. 29.5–54.8%) than self-report measures, respectively [6–8, 10].

Sleep quantity was similar, but sleep quality was better in this study, compared with rural Mozambicans [13]. Sri Lankan adults slept less than our sample, but at similar levels of sleep quality [34].

Untreated HIV + status was associated with poor sleep quality in rural Africans [4], which is somewhat in agreement with our results.

Sleep variables were not significantly and consistently related to adiposity. Self-report measures have found significant associations between TST and blood pressure [4, 6], insulin resistance [10] and adiposity [9].

The variability in South African sleep data is likely because of differences in social, cultural, economic and environmental factors [4, 10, 13]. Indeed, we found a direct relationship between sleep duration and SES, while Gomez-Olive et al. found an inverse relationship [4], highlighting the need for contextual, qualitative data. Our preliminary qualitative data has highlighted a number of themes which will require further investigation.

In conclusion, this report presents some of the first objectively-measured, free-living sleep data from a South African setting, and highlights the need to understand the contextual issues around sleep in this rural population.

## Limitations

Due to the small sample size and cross-sectional, convenience sampling in this study, the results cannot be readily generalized to the rural populations from whence the participants were recruited, nor can causality be shown.

## Supplementary information

**Additional file 1: Table S1.** Forced multiple linear regression models for anthropometric variables. **Table S2**. Backward-selection multiple linear regression models for anthropometric variables**. Table S3**. Forced multiple linear regression models for sleep variables. **Table S4**. Backward-selection multiple linear regression models for sleep variables.

**Additional file 2:** Informant_Consultation.docx. Qualitative feedback from the Informant Consultation interview.

## Data Availability

The dataset analysed during the current study is available from the corresponding author on reasonable request.

## Abbreviations

AWI-Gen: Africa Wits-INDEPTH partnership for Genomics studies
BMI: Body Mass Index
CI: Conicity Index
DHDSS: Dikgale Health and Demographic Surveillance System
NOA: Number of awakenings
PA: Physical activity
SE: Sleep efficiency
SFI: Sleep fragmentation index
TST: Total sleep time
WASO: Wake after sleep onset
