# Supplementary material for: Descriptive epidemiology of objectively-measured, free-living sleep parameters in a rural African setting
Source: BMC Res Notes. 2020 Jul 1;13:310. doi: 10.1186/s13104-020-05153-8 (PMC7329391; doi:10.1186/s13104-020-05153-8)
Supplement: Supplementary file 1 — Additional file 1: Table S1. Forced multiple linear regression models for anthropometric variables. Table S2. Backward-selection multiple linear regression models for anthropometric variables. Table S3. Forced multiple linear regression models for sleep variables. Table S4. Backward-selection multiple linear regression models for sleep variables. [file 13104_2020_5153_MOESM1_ESM.docx]

| Table S1. Forced multiple linear regression models for anthropometric variables | | | | | | | | |
| --- | --- | --- | --- | --- | --- | --- | --- | --- |
| Model | Goodness of fit | | Outcome | Factors | Model parameter | Significance | Correlations | Collinearity |
|  | adjusted R^2^ | *p* |  |  | β coefficient (95%CI) | *p* | Part | VIF |
| 1 | 0.3417 | <0.0001 | BMI | Age | -0.2 (-0.3; 0.01) | 0.0612 | -0.1277 | 1.2242 |
| (♀ + ♂, n = 145) | |  |  | Sex | -7.5 (-10.5; 4.5) | **<0.0001** | -0.3320 | 1.6919 |
|  |  |  |  | SES | +0.2 (-0.7; 1,1) | 0.6348 | 0.0322 | 1.1870 |
|  |  |  |  | FV intake | -0.9 (-2.3; 0.5) | 0.2079 | -0.0856 | 1.2356 |
|  |  |  |  | SSB intake | +0.9 (-1.2; 3.1) | 0.3993 | 0.0572 | 1.3160 |
|  |  |  |  | Tobacco usage | -0.04 (-2.5; 2.4) | 0.9731 | -0.0023 | 1.3597 |
|  |  |  |  | Alcohol usage | -1.5 (-4.9; 1.8) | 0.3660 | -0.0613 | 1.6617 |
|  |  |  |  | DM and/or HT | +3.8 (1.6; 6.0) | **0.0009** | 0.2305 | 1.1023 |
|  |  |  |  | HIV status | -4.3 (-7.0; -1.6) | **0.0021** | -0.2123 | 1.1537 |
|  |  |  |  | VMcpm | -0.0009 (-0.0035; 0.0017) | 0.5010 | -0.0456 | 1.4755 |
|  |  |  |  | TST | -0.01 (-0.02; 0.01) | 0.2659 | -0.0755 | 1.3857 |
|  |  |  |  | SE | +0.05 (-0.24; 0.34) | 0.7311 | 0.0233 | 1.4552 |
| 2 | 0.2798 | <0.0001 | WC | Age | -0.1 (-0.5; 0.2) | 0.4179 | -0.0510 | 1.2242 |
| (♀ + ♂, n = 145) | |  |  | Sex | -12.8 (-19.1; -6.4) | **0.0001** | -0.2813 | 1.6919 |
|  |  |  |  | SES | +0.6 (-1.2; 2.4) | 0.5177 | 0.0459 | 1.1870 |
|  |  |  |  | FV intake | -3.1 (-6.1; -0.1) | **0.0451** | -0.1431 | 1.2356 |
|  |  |  |  | SSB intake | +0.6 (-3.9; 5.1) | 0.7896 | 0.0189 | 1.3160 |
|  |  |  |  | Tobacco usage | -0.3 (-5.5; 4.8) | 0.9018 | -0.0087 | 1.3597 |
|  |  |  |  | Alcohol usage | +0.3 (-6.7; 7.4) | 0.9301 | 0.0062 | 1.6617 |
|  |  |  |  | DM and/or HT | +10.2 (5.6; 14.8) | **<0.0001** | 0.3094 | 1.1023 |
|  |  |  |  | HIV status | -8.5 (-14.6; -2.8) | **0.0039** | -0.2077 | 1.1537 |
|  |  |  |  | VMcpm | -0.003 (-0.009; 0.002) | 0.2227 | -0.0866 | 1.4755 |
|  |  |  |  | TST | -0.033 (-0.07; -0.001) | **0.0453** | -0.1429 | 1.3857 |
|  |  |  |  | SE | +0.3 (-0.3; 0.9) | 0.3233 | 0.0701 | 1.4552 |
| BMI = Body mass index (kg/m^2^), WC = Waist circumference (cm), Age = years, Sex (0 = female , 1 = male), SES = Socio-economic status (quintile), FV = Fruit and vegetable (tertiles: servings/day), SSB = Sugar-sweetened beverages (tertiles: servings/day), Tobacco and Alcohol usage (current user: 0 = No, 1= Yes), DM and/or HT = Diabetes Mellitus and/or Hypertension present (0 = negative, 1 = positive), HIV status (0 = negative, 1 = positive), VMcpm = average Vector Magnitude (counts/minute), TST = average Total sleep time (minutes/day), SE = average Sleep efficiency (%), VIF = variance inflation factor | | | | | | | | |

| Table S2. Backward-selection multiple linear regression models for anthropometric variables | | | | | | | | |
| --- | --- | --- | --- | --- | --- | --- | --- | --- |
| Model | Goodness of fit | | Outcome | Factors | Model parameter | Significance | Correlations | Collinearity |
|  | adjusted R^2^ | *p* |  |  | β coefficient (95%CI) | *p* | Part | VIF |
| 1 | 0.3580 | <0.0001 | BMI | Age | -0.2 (-0.3; -0.02) | **0.0309** | -0.1456 | 1.0776 |
| (♀ + ♂, n = 145) | |  |  | Sex | -7.7 (-10.0; -5.4) | **<0.0001** | -0.4365 | 1.0416 |
|  |  |  |  | DM and/or HT | +3.5 (1.4; 5.6) | **0.0012** | 0.2210 | 1.0453 |
|  |  |  |  | HIV status | -4.7 (-7.2; -2.2) | **0.0004** | -0.2434 | 1.0544 |
| 2 | 0.2804 | <0.0001 | WC | Sex | -10.5 (-15.5; -5.6) | **<0.0001** | -0.3000 | 1.0173 |
| (♀ + ♂, n = 145) | |  |  | DM and/or HT | +9.6 (5.1; 14.1) | **<0.0001** | 0.3002 | 1.0305 |
|  |  |  |  | HIV status | -9.6 (-14.9; -4.3) | **0.0005** | -0.2510 | 1.0146 |
| 3 | 0.1700 | <0.0001 | BMI | DM and/or HT | +4.9( 1.7; 7.2) | **0.0014** | 0.3100 | 1.0036 |
| (♀, n = 104) | |  |  | HIV status | -5.5 (-8.8; -2.2) | **0.0013** | -0.2973 | 1.0036 |
| 4 | 0.2584 | <0.0001 | WC | DM and/or HT | +12.5 (6.9; 18.1) | **<0.0001** | 0.3743 | 1.0391 |
| (♀, n = 104) | |  |  | HIV status | -11.6 (-18.3; -4.8) | **0.0011** | -0.2863 | 1.0316 |
|  | |  |  | FV intake | -4.1 (-7.6; -0.6) | **0.0218** | -0.1977 | 1.0566 |
|  | |  |  | VMcpd | -0.00004 (-0000008; 0.0000005) | 0.0797 | -0.1503 | 1.0276 |
| 5 | 0.1782 | 0.0163 | BMI | Employment | +3.1 (0.5; 5.6) | **0.0193** | 0.3505 | 1.0374 |
| (♂, n = 41) | |  |  | Alcohol usage | -2.4 (-4.6; -0.2) | **0.0349** | -0.3138 | 1.0180 |
|  |  |  |  | HIV status | -2.5 (-5.1; 0.1) | **0.0612** | -0.2767 | 1.0428 |
| 6 | 0.1702 | 0.0194 | WC | Education | +4.2 (-0.5; 8.8) | 0.0797 | 0.2596 | 1.0251 |
| (♂, n = 41) | |  |  | Tobacco usage | -6.3 (-12.2; -0.5) | **0.0342** | -0.3167 | 1.0159 |
|  |  |  |  | HIV status | -6.9 (-13.6; -0.1) | **0.0458** | -0.2977 | 1.0121 |
| BMI = Body mass index (kg/m^2^), WC = Waist circumference (cm), Age = years, Sex (0 = female , 1 = male), DM and/or HT = Diabetes Mellitus and/or Hypertension present (0 = negative, 1 = positive), HIV status (0 = negative, 1 = positive), FV = Fruit and vegetable (tertiles: servings/day), Education = highest level of education (0 = no formal education, 1 = primary, 2 = secondary, 3 = tertiary), Employment = Employment status (0 = unemployed, 1 = employed), Tobacco and Alcohol usage (current user: 1= Yes, 0 = No), VMcpd= average Vector Magnitude (counts/day), VIF = variance inflation factor | | | | | | | | |

| Table S3. Forced multiple linear regression models for sleep variables | | | | | | | | |
| --- | --- | --- | --- | --- | --- | --- | --- | --- |
| Model | Goodness of fit | | Outcome | Factors | Model parameter | Significance | Correlations | Collinearity |
|  | adjusted R^2^ | *p* |  |  | β coefficient (95%CI) | *p* | Part | VIF |
| 1 | 0.1156 | 0.0035 | TST | Age | +2.7 (0.7; 4.6) | **0.0069** | 0.2149 | 1.1784 |
| (♀ + ♂, n = 145) | |  |  | Sex | -34.4 (-68.7; -0.2) | **0.0488** | -0.1559 | 1.5794 |
|  |  |  |  | CI | -143.0 (-290.4; 4.4) | 0.0571 | -0.1504 | 1.2972 |
|  |  |  |  | Partnership | -9.5 (-29.0; 10.1) | 0.3403 | -0.0750 | 1.1053 |
|  |  |  |  | SES | +4.7 (-5.1; 14.4) | 0.3473 | 0.0739 | 1.0956 |
|  |  |  |  | Bedroom | +11.1 (-8.2; 30.3) | 0.2589 | 0.0889 | 1.1160 |
|  |  |  |  | Tobacco usage | -1.2 (-29.7; 27.3) | 0.9343 | -0.0065 | 1.3473 |
|  |  |  |  | Alcohol usage | +20.9 (-18.9; 60.7) | 0.3008 | 0.0814 | 1.6827 |
|  |  |  |  | DM and/or HT | -0.3 (-26.8, 26.2) | 0.9800 | -0.0020 | 11647 |
|  |  |  |  | HIV status | +13.7 (-17.8; 45.3) | 0.3901 | 0.0676 | 1.1356 |
|  |  |  |  | VMcpm | -0.1 (-0.1; -0.03) | **0.0002** | -0.3035 | 1.2650 |
| 2 | 0.1712 | 0.0001 | SE | Age | +0.1(-0.04; 0.2) | 0.2102 | 0.0955 | 1.1822 |
| (♀ + ♂, n = 145) | |  |  | Sex | +0.7 (-1.2; 2.5) | 0.4654 | 0.0555 | 1.6231 |
|  |  |  |  | CI | +1.3 (-6.5; 9.1) | 0.7356 | 0.0257 | 1.2877 |
|  |  |  |  | Partnership | -0.4 (-1.4; 0.6) | 0.4357 | -0.0593 | 1.1078 |
|  |  |  |  | SES | +0.5 (0.03; 1.0) | **0.0391** | 0.1580 | 1.0525 |
|  |  |  |  | Bedroom | +1.0 (0.1; 1.9) | **0.0257** | 0.1711 | 1.1195 |
|  |  |  |  | Tobacco usage | +0.4 (-1.1; 1.9) | 0.5693 | 0.0433 | 1.3455 |
|  |  |  |  | Alcohol usage | +0.7 (-2.4; 1.0) | 0.5040 | 0.0508 | 1.6877 |
|  |  |  |  | DM and/or HT | -0.6 (-2.0; 0.8) | 0.4280 | -0.0603 | 1.1764 |
|  |  |  |  | HIV status | -0.7 (-2.4; 1.0) | 0.4139 | -0.0622 | 1.1541 |
|  |  |  |  | VMcpm | -0.003 (-0.005; -0.002) | **<0.0001** | -0.3193 | 1.2603 |
| TST = average Total sleep time (minutes/day), SE = average Sleep efficiency (%), Age = years, Sex (0 = female , 1 = male), CI = Conicity Index, Partnership = Partnership status (0 = never married/cohabited, 1 = married/living with partner, 2 = divorced/widowed), SES = Socio-economic status (quintile), Bedroom = people-to-bedroom density, Tobacco and Alcohol usage (current user: 0 = No, 1= Yes), DM and/or HT = Diabetes Mellitus and/or Hypertension present (0 = negative, 1 = positive), HIV status (0 = negative, 1 = positive), VMcpm = average Vector magnitude (counts/minute), VIF = variance inflation factor | | | | | | | | |

| Table S4. Backward-selection multiple linear regression models for sleep variables | | | | | | | | |
| --- | --- | --- | --- | --- | --- | --- | --- | --- |
| Model | Goodness of fit | | Outcome | Factors | Model parameter | Significance | Correlations | Collinearity |
|  | adjusted R^2^ | *p* |  |  | β coefficient (95%CI) | *p* | Part | VIF |
| 1 | 0.1352 | <0.0001 | TST | Age | +2.0 (0.1; 3.8) | **0.0340** | 0.1659 | 1.0763 |
| (♀ + ♂, n = 145) | |  |  | Bedroom | +15.6 (-0.3; 31.5) | 0.0541 | 0.1505 | 1.0186 |
|  |  |  |  | CI | -145.2 (-276.8; -13.6) | **0.0308** | -0.1691 | 1.0583 |
|  |  |  |  | VMcpm | -0.05 (-0.1; -0.2) | **0.0002** | -0.2966 | 1.0377 |
| 2 | 0.1722 | 0.0023 | SE | SES | +0.5 (0.3; 1.0) | **0.0389** | 0.1580 | 1.0408 |
| (♀ + ♂, n = 145) | |  |  | Bedroom | +1.1 (0.2; 1.9) | **0.0171** | 0.1829 | 1.0398 |
|  |  |  |  | VMcpm | -0.004 (-0.005; -0.003) | **<0.0001** | -0.4028 | 1.0235 |
| 3 | 0.1854 | 0.0001 | TST | Parity | +12.2 (6.1; 18.2) | **0.0001** | 0.3551 | 1.0551 |
| (♀, n = 104) | |  |  | CI | -164.5 (-295.0; -34.0) | **0.0140** | -0.2225 | 1.2135 |
|  |  |  |  | SES | +11.5 (1.8; 21.3) | **0.0203** | 0.2098 | 1.1631 |
|  |  |  |  | Education | -23.2 (-44.7; -1.6) | **0.0354** | -0.1898 | 1.3161 |
|  |  |  |  | VMcpd | -0.00003 (-0.00005; -0.000006) | **0.0005** | -0.3288 | 1.0069 |
| 4 | 0.1378 | 0.0002 | SE | Parity | +0.4 (0.01; 0.8) | **0.0430** | 0.1875 | 1.0069 |
| (♀, n = 104) | |  |  | VMcpd | -0.000002 (-0.0001; -0.00002) | **0.0005** | 0.3288 | 1.0069 |
| 5 | 0.1908 | 0.0025 | TST | VMcpd | -0.00006 (-0.0001; -0.00002) | **0.0025** | -0.4594 | - |
| (♂, n = 41) | |  |  |  |  |  |  |  |
| 6 | 0.2000 | 0.0054 | SE | SES | +1.3 (0.3; 2.3) | **0.0117** | 0.3746 | 1.0645 |
| (♂, n = 41) | |  |  | VMcpd | -0.000002 (-0.000004; -0.0000007) | **0.0077** | -0.3983 | 1.0645 |
| TST = average Total sleep time (minutes/day), SE = average Sleep efficiency (%), Age = years, Bedroom = people-to-bedroom density, CI = Conicity Index, VMcpm = average Vector Magnitude (counts/minute), VMcpd = average Vector Magnitude (counts/day), SES = Socio-economic status (quintile), Education = highest level of education (0 = no formal education, 1 = primary, 2 = secondary, 3 = tertiary), Parity = number children, VIF = variance inflation factor | | | | | | | | |
