# Supplementary material for: Descriptive epidemiology of objectively-measured, free-living sleep parameters in a rural African setting
Source: BMC Res Notes. 2020 Jul 1;13:310. doi: 10.1186/s13104-020-05153-8 (PMC7329391; doi:10.1186/s13104-020-05153-8)
Supplement: Supplementary file 2 — Additional file 2: Informant_Consultation.docx. Qualitative feedback from the Informant Consultation interview. [file 13104_2020_5153_MOESM2_ESM.docx]

| Item | Result | | Comment/Feedback |
| --- | --- | --- | --- |
| Figure 1A-B | Total sleep time:  Weekday>Weekend (*p*≤0.0409) | | Very active on Fridays to Saturdays. Many community activities: funerals, *shebeens* (informal licensed drinking place), entertainment, marriages etc. Monday people are tired and recovery occurs on Tuesday and Wednesday |
| Figure 1C | Number of awakenings:  Weekday>Weekend (*p*≤0.0451) | | People deliberately try to sleep long longer during weekdays to recover from the weekend– they are not necessarily tired. This is not the usual sleep pattern |
| Table 1 | Sugar sweetened beverages:  Female>Male, *p*=0.0050 | | While males use traditional beer, the preferred drink of females is fruit and soda juices (in this age group) |
| Table 2 | Nocturnal sleep time:  Female>Male, *p*=0.0463 | | Males come home later, females go to bed tired (house work, child care, cooking) |
|  | In bed time:  Weekday>Weekend, *p*=0.0016 | | Males generally have no home responsibilities; start with early sleep ie get home and nap or fall asleep in front of TV. They will get up/wait until food ready etc |
|  | Sleep Fragmentation Index:  Weekday>Weekend, *p*=0.0458 | | There are fewer concerns around childcare etc on weekend. Many do not work on the weekends, and are more relaxed |
| Table S1* | Waist circumference (outcome):  Fruit-vegetable intake (factor: β=-3.1, *p*=0.0451) | | Those that eat more fruit and vegetables, eat less *pap* (mielie meal, mielie pap, traditional porridge) and rice. Those who eat more meat, eat more *pap* and rice. |
| Table S2* | Waist circumference/Body mass index (outcomes) | Employment (factor: β=+3.1, *p*=0.0193) | Males have more money to spend on street food etc |
|  |  | Alcohol use (factor: β=-2.4, *p*=0.0349) | Males who use/abuse alcohol do not eat enough/healthily and are thin |
|  |  | Tobacco use (factor: β=-6.3, *p*=0.0342) | Males generally use tobacco and smoking reduces hunger, and users are thin |
|  |  | Education (factor: β=+4.2, *p*=0.0797) | With better education males have a higher socio-economic status ie can access certain foods etc |
|  |  | Vector magnitude (factor: β=-0.00004, *p*=0.0797) | Females are generally involved in a substantial subsistence lifestyle which is hard, with busy long hours, and is stressful |
| Table S3* | Total sleep time (outcome) | Age (factor: β=+2.7, *p*=0.0069) | There are less responsibilities in the lives of older people eg children |
|  | Sleep Efficiency (outcome) | Bedroom (factor: β=+1.0, *p*=0.0257) | Difficult to explain - people do feel safer with more people in the house at night |
| Table S4* | Total sleep time (outcome) | Education (factor: β=-23.2, *p*=0.0354) | Difficult to explain - might be that females in this age group are very involved in Adult Basic Education Training (ABET), and the training might displace home responsibilities so they sleep less/go to bed later so that can finish home responsibilities or stay up later so they can study |
|  | Sleep Efficiency (outcome) | Parity (factor: β=+0.4, *p*=0.0430) | The children in the house are older and thus are able to help females with house work etc |

*See Additional File 2
